# Na^+^/H^+^ exchanger (NHE) in Pacific white shrimp (*Litopenaeus vannamei*): Molecular cloning, transcriptional response to acidity stress, and physiological roles in pH homeostasis

**DOI:** 10.1371/journal.pone.0212887

**Published:** 2019-02-27

**Authors:** Hongmei Li, Chunhua Ren, Xiao Jiang, Chuhang Cheng, Yao Ruan, Xin Zhang, Wen Huang, Ting Chen, Chaoqun Hu

**Affiliations:** 1 CAS Key Laboratory of Tropical Marine Bio-resources and Ecology (LMB) South China Sea Institute of Oceanology, Chinese Academy of Sciences, Guangzhou, Guangdong, China; 2 University of the Chinese Academy of Sciences, Beijing, China; 3 South China Sea Bio-Resource Exploitation and Utilization Collaborative Innovation Center, Guangzhou, Guangdong, China; Shanghai Ocean University, CHINA

## Abstract

Na^+^/H^+^ exchangers are the most common membrane proteins involved in the regulation of intracellular pH that concurrently transport Na^+^ into the cells and H^+^ out of the cells. In this study, the full-length cDNA of the Na^+^/H^+^ exchanger (*NHE*) from the Pacific white shrimp (*Litopenaeus vannamei*) was cloned. The *LvNHE* cDNA is 3167 bp long, contains a 5’-untranslated region (UTR) of 74 bp and a 3’-UTR of 456 bp and an open reading frame (ORF) of 2637 bp, coding for a protein of 878 amino acids with 11 putative transmembrane domains and a long cytoplasmic tail. *Lv*NHE shows high sequence homology with mud crab NHE at the amino acid level. *LvNHE* mRNA was detected in the hepatopancreas, gill, eyestalk, skin, heart, intestine, muscle, brain and stomach, with the highest abundance in the intestine. In the shrimp intestinal fragment cultures exposed to gradually declining pH medium (from pH 8.0 to pH 6.4), the *LvNHE* mRNA expression was significantly stimulated, with the highest response when incubated in pH 7.0 medium for 6 h. To investigate the functional roles of *Lv*NHE in pH regulation at the physiological and cellular levels, the *LvNHE* mRNA expression was silenced by siRNA knockdown. Upon low-pH challenge, the hemolymph pH was significantly reduced in the *LvNHE* mRNA knockdown shrimp. In addition, knockdown of *LvNHE* mRNA reduced the recovery capacity of intracellular pH in intestinal fragment cultures after acidification. Altogether, this study demonstrates the role of *NHE* in shrimp response to low pH stress and provides new insights into the acid/base homeostasis mechanisms of crustaceans.

## Introduction

The Pacific white shrimp, *Litopenaeus vannamei*, is a penaeid shrimp naturally distributed along the Pacific coast of the Americas from northern Mexico to northern Peru [1]. *L*. *vannamei* was introduced to East Asia in 1985 and has become one of the major cultured crustacean species in this region [2, 3]. Under a high-density culture condition, the acidity/alkalinity (pH value) of aquatic environments fluctuates frequently due to acid rain, organic residue decomposition and carbon dioxide release, which may give rise to a harmful stress to *L*. *vannamei* [4, 5]. The shrimp may suffer physiological damage, such as suppressed immune activity [6], induced respiratory burst [7] and disordered ion balance, which consequently result in slow growth, abnormal behaviors and increased mortality [8].

It has been shown that pH is an important factor affecting crustacean life [9]. During the changes in environmental pH, the intracellular pH (pHi) in aquatic crustaceans may be kept stable to maintain an appropriate environment for cellular activities. *L*. *vannamei* can adapt to the change in pH in culture and continue to live and function mainly by transporting ion and water molecules across the cell membrane [8, 10, 11]. Several ion transport-related proteins have been demonstrated in *L*. *vannamei* with their functions in salinity and/or pH homeostasis, such as Na^+^/K^+^-ATPase (NKA) [12, 13], carbonic anhydrase (CA) [14], V-type H^+^ ATPase (VHA) [15] and Na^+^/HCO_3_^-^ cotransporter (NBC) [16].

The sodium/proton exchanger (Na^+^/H^+^ exchanger or NHE), a member of the solute carrier (SLC) 9A family that belongs to the cation/proton antiporters (CPA) superfamily, is a membrane ion transport-related protein that concurrently transports Na^+^ into the cell and H^+^ out of the cell [17]. The *NHE* gene was first isolated from the small intestine and kidney of rat [18]. After the first *NHE* gene was cloned, at least nine more functional mammalian *NHE* genes were subsequently identified and named *NHE*1-9 (*SLC*9A1-9) [17]. The NHE isoforms contain a similar topological structure with 11–13 transmembrane (TM) domains at the N-terminus for ion exchange, and the C-terminus of NHE is a cytoplasmic regulatory region. In most types of animal cells, NHE localizes in the cytoplasmic membrane and plays important roles in regulating intracellular pH, and it is involved in cell volume regulation, transepithelial absorption and electrolyte secretion [19]. In cells at a physiological pH_i_, the basal activity of NHE is very low. Upon a decrease in pH_i_, the NHE activity sharply increases to adjust the acidified pH_i_ by rapidly extruding protons in exchange for extracellular Na^+^. In humans, NHE is involved in several pathophysiologic processes such as ischemia, hypertrophy, hypertension and arrhythmias [20].

Mediation of pH_i_ by NHE is one of the most ubiquitous and important mechanisms in cell recovery after an acid pulse [21]. The NHE cDNA has also been identified in aquatic animals including seawater fishes sculpin and mummichog [22], trout [23] and lobster [24]. However, NHE has not been investigated in *L*. *vannamei* to date. To illustrate the potential roles of NHE in the regulation of acid-base homeostasis in this widely cultured economic species, in this study, the full-length *NHE* cDNA (designated *LvNHE*) was first isolated from the *L*. *vannamei* intestine. The protein structure and tissue expression profile of *Lv*NHE were further investigated. The change in *LvNHE* transcript levels in the intestinal fragment culture was analyzed after exposure of culture medium to gradually decreasing pH. The functions of *Lv*NHE were further investigated by measuring the capacity of pH regulation in the hemolymph and the rates of pH_i_ recovery in intestinal fragments after RNA interference (RNAi).

## Materials and methods

### Animals and sample collection

For the studies of molecular cloning, tissue distribution, intestine fragment culture and hemolymph collection, healthy *L*. *vannamei* about 3-months old with body lengths of 7.0–9.0 cm and body weights of 9.0–13.0 g were obtained from the Jinyang Shrimp Culture Center, Maoming, China. Shrimp were acclimated for one week at 28±0.5°C in tanks containing aerated seawater (salinity 30‰ and pH 8.0) and fed commercial shrimp feed twice daily until 24 h before the experiments began. The hepatopancreas, gill, eyestalk, skin, heart, intestine, muscle, brain and stomach were collected, frozen immediately in liquid nitrogen and stored at -80°C for further studies.

### Molecular cloning and sequence analysis

Total RNA from the *L*. *vannamei* intestine was extracted with the RNA Extraction Kit (TianGen) and reverse-transcribed into the first-strand cDNA using the PrimeScript RT Kit (TaKaRa). Primers for *LvNHE* cDNA cloning (shown in Table 1) were designed based on the sequence of a transcriptome from *L*. *vannamei* previously constructed in our laboratory [25]. To obtain the full-length cDNA sequence of *LvNHE*, 3'- and 5'-rapid amplification of cDNA ends (RACE) was applied. The amino acid sequence, protein molecular weight (MW) and isoelectric point (pI) of *Lv*NHE were predicted using Lasergene 5.1 (DNASTAR, Inc.). The functional sites and TM domains of *LvNHE* were deduced using the PROSITE program (http://www.expasy.org/prosite) and TMHMM Server v. 2.0 (http://www.cbs.dtu.dk/services/TMHMM/), respectively.

**Table 1:** Primers and siRNA sequences used in this study.

Name	Sequence (5'-3')
**For cDNA cloning**	
3' RACE1	TCTGTGGGTTTACACAATGCA
3' RACE2	AGGCTGGTGCATTGAGTAGTTT
5' RACE1	AGGGTAAGCGACACCAACAC
5' RACE2	CACCGGAAGGACATGAGGT
C-NHEa-F	CACTGTGCTGGATATCTGCAATGTGGAGAGCATGGGCGT
C-NHEa-R	TGGTCTAACATGTTGGCCAGAGAGAA
C-NHEb-F	CTGGCCAACATGTTAGACCAAACAATTGACCCAAGGAGGA
C-NHEb-R	AGTCCAGTGTGGTGGAATTCTCAAACCACATCCTCATTTTCTGA
**For qPCR**	
Q*Lv*NHE-F	GGCGGAGCTCTTTCACTTCTC
Q*Lv*NHE-R	GGTGCCAGATATGGTCTTTGC
Qβ-actin-F	CCGGCCGCGACCTCACAGACT
Qβ-actin-R	CCTCGGGGCAGCGGAACCTC
**For RNAi**	
siRNA-1 sense	GCAUCCACCUCAUGUCCUUTT
siRNA-1 antisense	AAGGACAUGAGGUGGAUGCTT
siRNA-2 sense	GCUUUAUUCUCUGGACAAUTT
siRNA-2 antisense	AUUGUCCAGAGAAUAAAGCTT
siRNA-3 sense	CCCGUCUUCCUGUAUCCAATT
siRNA-3 antisense	UUGGAUACAGGAAGACGGGTT
NC siRNA sense	UUCUCCGAACGUGUCACGUTT
NC siRNA antisense	ACGUGACACGUUCGGAGAATT

### Tissue distribution of *LvNHE* mRNA

RNA extracted from the hepatopancreas, gill, eyestalk, skin, heart, intestine, muscle, brain and stomach of three shrimp was reverse-transcribed into first strand cDNAs using the PrimeScript RT Kit with gDNA Eraser (Takara). The gene-specific primers Q*LvNHE*-F and Q*LvNHE*-R (Table 1) were designed based on the obtained cDNA sequences, and the expression pattern of *LvNHE* mRNA was detected by real-time PCR using SYBR Premix Ex Taq^™^ II Kit (TaKaRa) by the following procedure: 40 cycles of 5 seconds at 95°C for denaturation and 30 seconds at 60°C for annealing, extension and signal capture. In this case, *β-actin* was used as an internal control amplified with the specific primers Qβ-actin-F and Qβ-actin-R designed based on *L*. *vannamei β-actin* cDNA sequence (Table 1). The relative expression levels of *LvNHE* were calculated using the comparative Ct method with the formula 2^-ΔΔCt^.

### pH challenge in intestine fragment culture

Intestines were removed from shrimp under sterile conditions. The excrements were discarded, and intestines were washed in PBS containing 1 mg/ml streptomycin and 1000 IU/ml penicillin for 5 min. After rinsing 5 times with PBS, intestines were cut into 3-mm pieces. The fragments from 10 shrimp were mixed together, and 12 ml of Sf-900 II cell culture medium (Sf-900 II SFM, ThermoFisher) containing 5% fetal bovine serum (Gibco), 1 mg/ml streptomycin and 1000 IU/ml penicillin was added. The fragments were sequentially passed through 100 μM and 40 μM cell sieves, plated in 12-well plates (Corning) and cultured at 28°C for 12 h [26, 27]. Then, the intestinal fragments were collected by centrifugation at 1000 rpm for 3 min, and the supernatants were discarded. The fragment resuspensions were treated with gradient pH 6.4–8.0 medium for 2, 6 and 12 h. In this case, the fragments were independently cultured in three wells for each pH at each time point, and the mRNA levels of *LvNHE* were detected by real-time PCR as described above.

### RNAi-mediated *LvNHE* gene silencing

Small interfering RNAs (siRNAs) were designed using the siDirect version 2.0 online program (http://sidirect2.rnai.jp/). siRNA-1, siRNA-2, and siRNA-3 designed to target *LvNHE* mRNA and a nontargeting siRNA (NC-siRNA) used as a negative control (Table 1) were synthesized by Sangon Biotech Company and dissolved in DEPC-H_2_O. Shrimp about 1-month old with body lengths of 5.12±0.61 cm and body weights of 3.55±0.82 g were used for RNAi experiments. To confirm the interference efficiencies of the synthesized siRNAs, three siRNAs (siRNA-1, siRNA-2 and siRNA-3) were injected at the concentration of 1 μg/g body weight (bwt). In this case, DEPC-H_2_O and NC-siRNA were injected as the control groups. Shrimp were cultured at 28°C in the tanks containing aerated seawater (salinity 30‰, pH 8.0). Intestines from 3 individuals in each group were respectively collected 6 and 12 h after injection. The expression levels of *LvNHE* were measured by real-time PCR as described above.

### Measurement of hemolymph pH

Shrimp were randomly divided into 10 groups and cultured in 100-L independent tanks. Shrimp injected with *LvNHE* siRNA (n = 29 for 6 h and n = 25 for 12 h), DEPC-H_2_O (n = 26 for 6 h and n = 24 for 12 h) and NC-siRNA (n = 21 for 6 h and n = 28 for 12 h), or without injection (n = 24 for 6 h and n = 25 for 12 h) were cultured in pH 5.8-acidified seawater for 6 h or 12 h. In this case, shrimp cultured in pH 8.0 normal seawater for 6 h (n = 19) or 12 h (n = 20) were used as the control groups. Approximately 0.2 ml of hemolymph was collected from the ventricles of the shrimp and centrifuged at 1000 g for 3 min. The supernatants were transferred to a new EP tube, and the hemolymph pH levels were determined by using a micro pH electrode (P13, Bante Instruments) connected to a pH analyzer (SevenEasy FE20, Mettler Toledo).

### Measurement of intracellular pH in intestine

The intracellular pH of shrimp intestinal fragment cultures was monitored using the pH-sensitive dye 2',7'-bis(carboxyethyl)-5(6)-carboxyfluorescein-pentaacetoxymethyl ester (BCECF-AM) (B1150, Invitrogen) as described previously [28]. The fluorescence was measured by using a Synergy H1 fluorescence spectrometer (BioTek) with an emission wavelength of 535 nm and excitation wavelengths of 440 nm and 490 nm. The calibration curve for the pH_i_ signal was constructed by the high potassium-nigericin technique [29, 30]. Intracellular pH was acidified using the NH_4_Cl (20 mM) perfusion technique [28]. Briefly, after the excrements were removed, shrimp intestine was cut into pieces of 1.0×1.0×1.0 mm^3^ in size with a McILwain tissue chopper (Ted Pella), and fragments of approximately 30 mg per well were placed into the 96-well plate. The intestine fragments were incubated with Na^+^-free salt solution (Na^+^ was replaced by N-methyl-D-glucamine), then perfused with a physiological salt solution containing 20 mM NH_4_Cl (NaCl was replaced by NH_4_Cl) for more than 10 min, which was then switched to the NH_4_Cl free and Na^+^-free salt solution to produce an acid load [31, 32]. Approximately 5 min later, extracellular Na^+^ was introduced to initiate Na^+^/H^+^ exchanger-mediated intracellular pH recovery. The physiological salt solution contained the following (in mM): NaCl (125), KCl (5), MgSO_4_ (1.2), CaCl_2_ (1), KH_2_PO_4_ (2), glucose (5), and Hepes (32.2), pH 7.4. The rates of pHi recovery (dpH/dt) were determined within 0 to 5 min following the addition of extracellular Na^+^. The pHi of intestinal fragments and the pH recovery rate of the *LvNHE* knockdown intestinal fragments were measured by using the method described above.

### Statistical analysis

Data are expressed as the mean±SE and were analyzed with Student’s *t*-test or one-way ANOVA followed by Fisher’s least significant difference (LSD) test by using SPSS (IBM Software).

## Results

### Molecular cloning and sequence analysis of *LvNHE*

**Fig 1:**
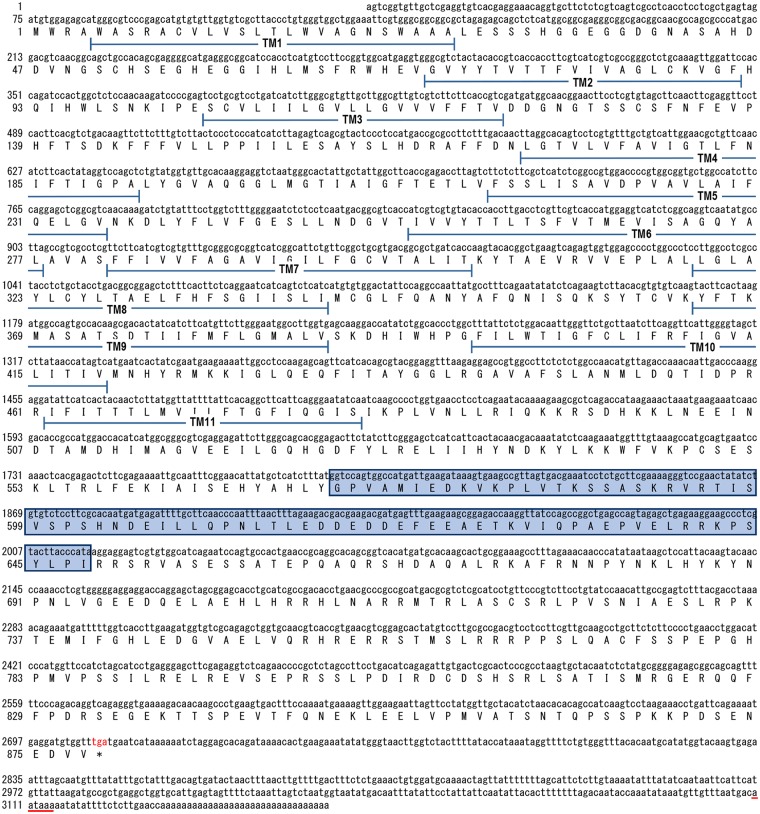
The full-length cDNA sequence and its deduced amino acid sequence of *Lv*NHE. The start codon (ATG) and stop codon (TGA) are shown in red. The transmembrane (TM) domains are indicated. The NEXCaM regulatory region is boxed. The consensus polyadenylation (AATAAA) signal is underlined.

**Fig 2:**
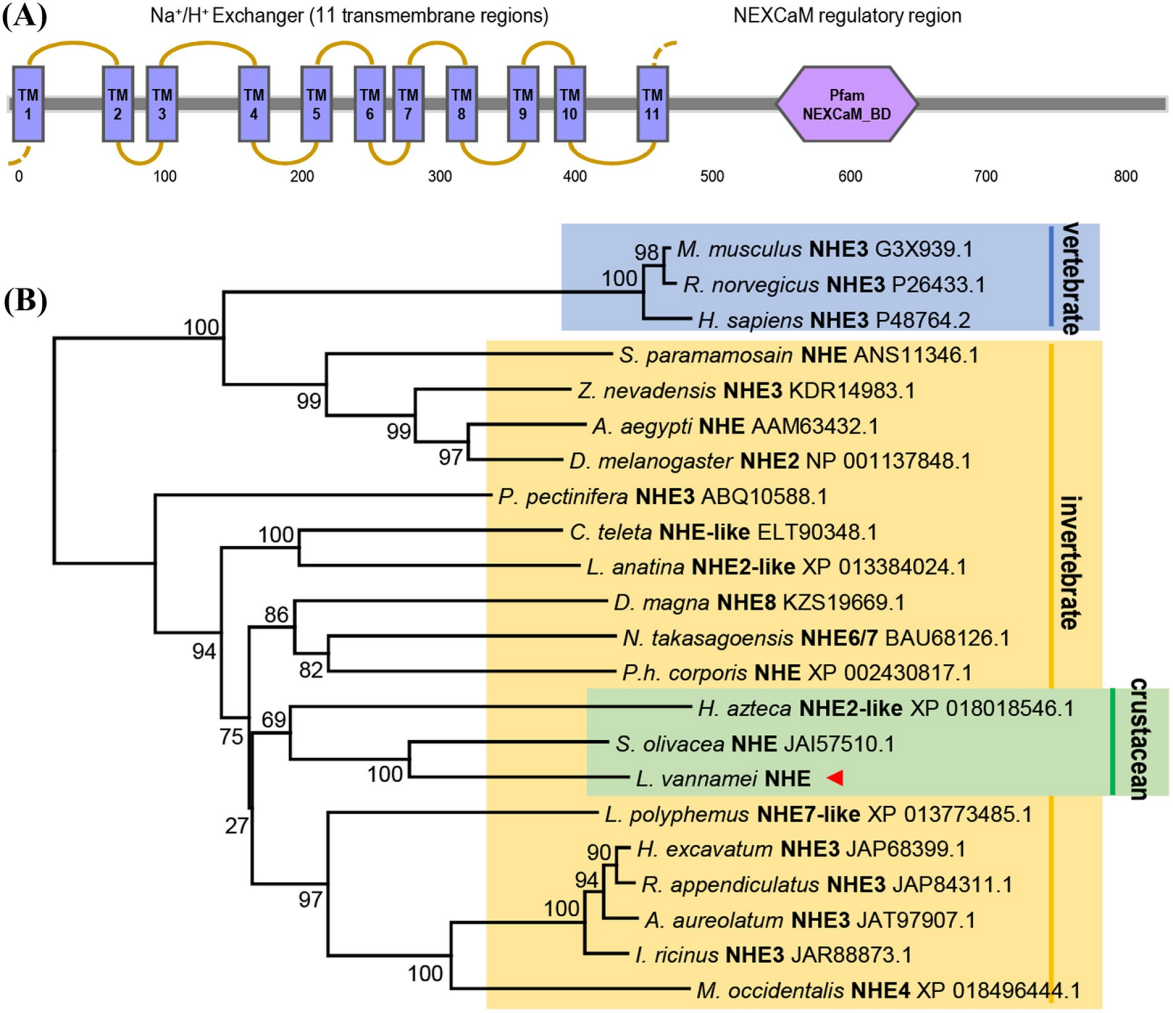
Structural domains of *LvNHE* and phylogenetic analysis. (A) Structural domains of *LvNHE* predicted by using the SMART program. The 11 transmembrane (TM) domains and the NEXCaM_BD feature with respective locations are indicated. (B) Phylogenetic analysis of NHEs in various species by using the Neighbor-Joining method with a bootstrap value of 1000. The newly identified *Lv*NHE is marked by a triangle.

The full-length cDNA sequence of *LvNHE* was obtained by the 3’-/5’-RACE approach. The *LvNHE* cDNA is 3167 bp long, contains a 5’-untranslated region (UTR) of 74 bp, a 3'-UTR of 456 bp and an open reading frame (ORF) of 2637 bp that encodes a protein of 878 amino acids. A typical polyadenylation signal (TATAAA) is located 40 bp upstream of the poly-A tail (Fig 1). The deduced molecular weight of *Lv*NHE was 98 kDa, the predicted isoelectric point was 6.27, and transmembrane domains were predicted by TMHMM Server (Fig 2A).

By phylogenetic analysis of NHEs from different animal species, our newly cloned *Lv*NHE (GenBank No. MK111428) has the shortest evolutionary distance from that of the mud crab (*Scylla olivacea*) and is clustered with other crustacean NHEs, insect NHEs, and human NHE3 (Fig 2B).

### Tissue expression pattern and pH-induced expression of *LvNHE*

**Fig 3:**
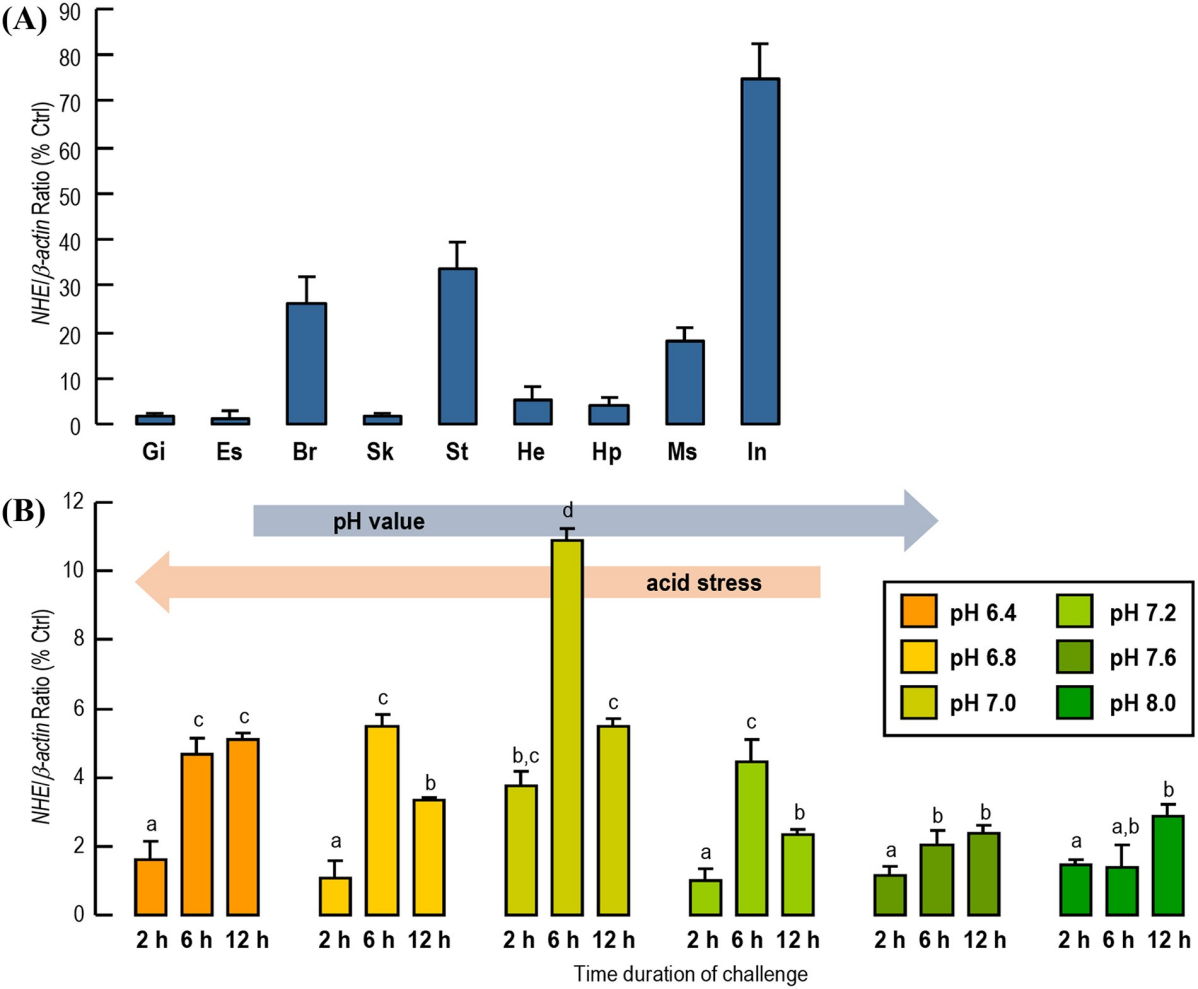
Tissue expression pattern and pH-induced expression of *LvNHE*. (A) Expression profiles of *LvNHE* mRNA in various *L*. *vannamei* tissues, including the hepatopancreas (Hp), gill (Gi), eyestalk (Es), skin (Sk), heart (Ht), intestine (In), muscle (Ms), brain (Br) and stomach (St). (B) *LvNHE* expression levels in the intestines after low pH challenge. The *LvNHE* mRNA levels were normalized by *β-actin* expression. The data are expressed as the mean±SE (n = 3), and experimental groups denoted by the same letter represent a similar level (*P*>0.05, ANOVA followed by Fisher’s LSD test).

Quantitative real-time PCR was used to detect *LvNHE* expression in multiple tissues. The results showed that *LvNHE* transcripts were highly expressed in the intestine, stomach, brain, and muscle, with the highest expression in the intestine (Fig 3A).

The shrimp intestinal fragments were exposed to the pH 6.4–8.0 gradient in the medium for 2 h, 6 h, and 12 h, and mRNA levels of *LvNHE* were detected by real-time PCR. The *NHE* mRNA expression in intestinal fragments significantly increased when incubated at pH 7.0 (Fig 3B). In culture medium at pH lower than 7.2, the *NHE* expression increased significantly from 2 h to 6 h, but in culture medium at pH higher than 7.2, the *NHE* expression level changed little with time, implying that the culture environment below pH 7.2 probably causes acidification of the intracellular environment and induces *LvNHE* expression.

### Effect of *LvNHE* mRNA knockdown on the hemolymph pH upon low pH challenge

**Fig 4:**
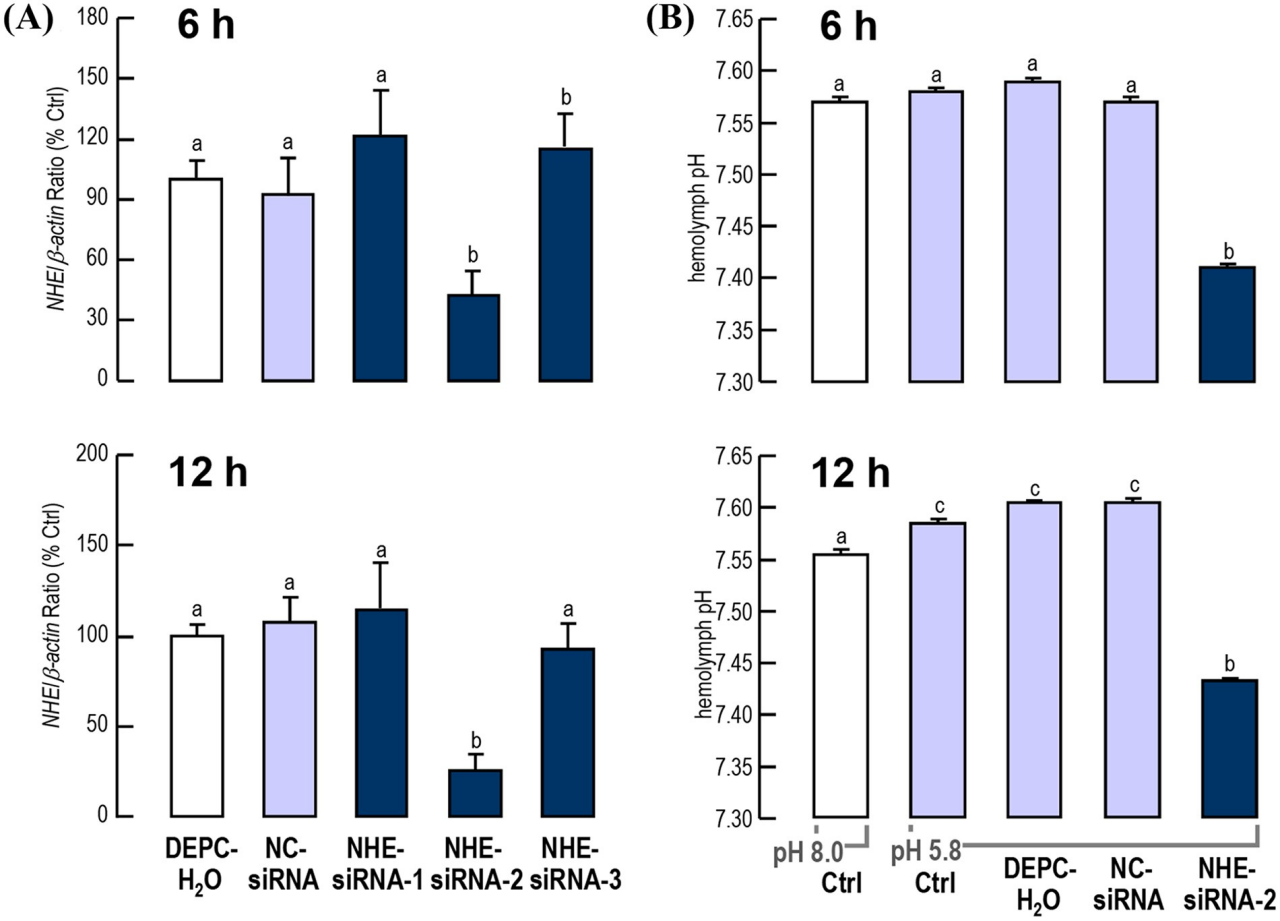
Effect of *LvNHE* mRNA knockdown on the hemolymph pH upon low pH challenge. (A) Expression of *LvNHE* transcripts in shrimp intestines after injection of DEPC-H_2_O, NC-siRNA and *LvNHE* siRNA-1, -2 and -3 at 6 and 12 h. (B) The hemolymph pH of *LvNHE*-siRNA-injected shrimp upon low (5.8) pH challenge for 6 and 12 h. In this case, the dosages for all siRNA groups were 1 μg/g bwt, and the individual numbers for all groups were 3. For mRNA and hemolymph pH measurements, the data are expressed as the mean±SE, and experimental groups denoted by the same letter represent a similar level (*P*>0.05, ANOVA followed by Fisher’s LSD test).

To understand the regulatory effects of *Lv*NHE on hemolymph pH, shrimp were injected with 1 μg/g bwt siRNA-1, -2, -3, NC-siRNA or DEPC-H_2_O. The results showed that siRNA-2 had the highest RNAi efficiency, especially 12 h after injection when more than 70% of *LvNHE* mRNA was degraded (Fig 4A). At 6 h after challenge, the hemolymph pH of the pH 8.0 control, pH 5.8 control, pH 5.8 DEPC-H_2_O-injected and NC-siRNA injected and *LvNHE* siRNA-2 injected groups were 7.57±0.005, 7.58±0.004, 7.59±0.003, 7.57±0.004 and 7.41±0.004, respectively (Fig 4B). At 12 h after challenge, the hemolymph pH of the pH 8.0 control, pH 5.8 control, pH 5.8 DEPC-H_2_O-injected and NC-siRNA injected and *LvNHE* siRNA-2 injected groups were 7.56±0.005, 7.59±0.004, 7.61±0.002, 7.61±0.003 and 7.44±0.003, respectively (Fig 4B).

### Effect of *LvNHE* mRNA knockdown on pH_i_ recovery in intestinal fragment culture

**Fig 5:**
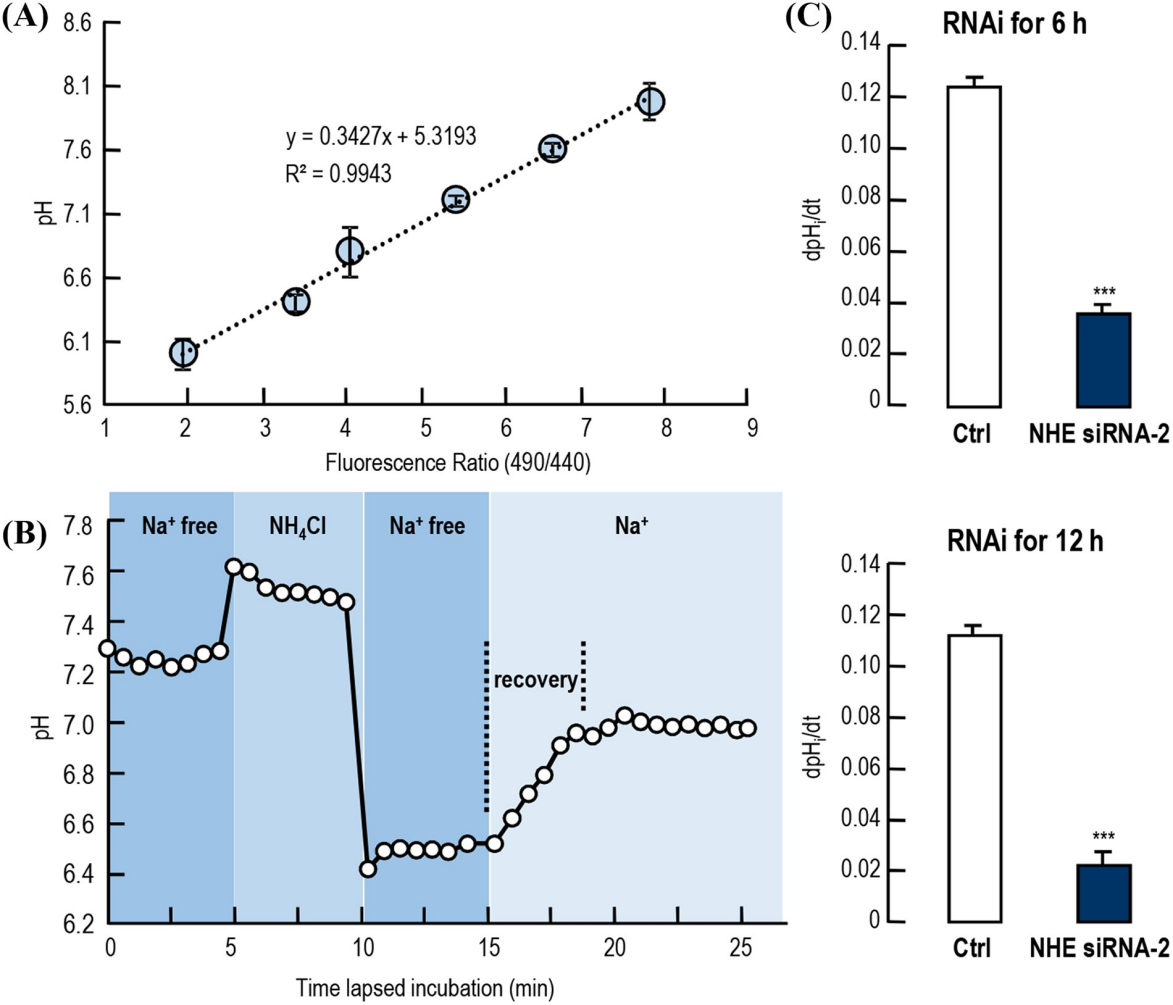
Effect of *LvNHE* mRNA knockdown on pH_i_ recovery in intestinal fragment culture. (A) The calibration curve for transforming the fluorescence ratio (490/440) into a pH value. (B) pH_i_ recovery in intestinal fragment culture following intracellular acidification upon readdition of Na^+^. Intracellular pH was measured, and an acid load was applied using the NH_4_Cl-prepulse technique in the absence of extracellular Na^+^. (C) The pHi recovery rate (dpH/dt) of intestinal fragment culture from shrimp injected with *LvNHE* siRNA-2 for 6 and 12 h. The data for calibration curve and pHi recovery rate are expressed as the mean±SE (n = 8), and experimental groups denoted by the same letter represent a similar level (*P*>0.05, ANOVA followed by Fisher’s LSD test).

In this study, the pH_i_ recovery rates after acidification in the control and NHE-knockdown shrimp intestinal fragment cultures were determined. The pH-dependent fluorescence ratio (490 nm/440 nm) was calculated with a high K^+^ gradient pH solution, and the calibration curve was constructed (Fig 5A). In the intestinal fragments without prior intracellular acidification by NH_4_Cl, the basal pH was 7.26±0.054. Then, an ammonium bath caused alkalization, and when ammonium was removed to return to Na^+^-free buffer, a remarkable acidification was observed. However, after the addition of Na^+^ to the bath solution, a Na^+^-dependent pH_i_ recovery was observed (Fig 5B). Records were obtained from two separate experiments. In the control intestinal fragments, after Na^+^ introduction, cells started to recover toward their normal pH at a rate of 0.123±0.005 (for 6 h control) or 0.111±0.004 (for 12 h control) pH units per min. In the *LvNHE*-knockdown intestinal fragments, the dpH/dt values for the 6 h group and 12 h group were 0.035±0.004 and 0.021±0.005, respectively (Fig 5C and 5D). These results showed that the recovery rates in *LvNHE*-knockdown intestinal fragments were significantly lower than in the control groups, indicating that the *LvNHE*-interference affects the realkalization process after acidification.

## Discussion

Numerous studies have shown that NHEs play important roles in ion transport in animals [33]. In the present study, a full-length cDNA encoding *NHE* from *L*. *vannamei* was first cloned. Sequence analysis revealed that *Lv*NHE contained 11 TM domains at the N-terminus and a NEXCaM regulatory region at the C-terminus, with a coiled-coil structure for binding of the Ca^2+^/calmodulin complex [34]. To assess the molecular evolutionary relationship between *Lv*NHE and its counterparts in other species, a phylogenetic tree was constructed and revealed a high diversity in the family of Na^+^/H^+^ exchangers (Fig 2B). Additionally, phylogenetic analysis further indicated that *Lv*NHE was clustered with the NHEs from other crustacean species, such as mud crab.

The tissue distribution results showed that *LvNHE* was expressed most abundantly in the intestine of *L*. *vannamei* (Fig 3A). Osmotic and ionic regulation in crustaceans is mostly accomplished by the multifunctional gills, together with the excretory and digestive organs [35, 36]. Our results provided evidence that the intestine is one of the most important organs for pH regulation in shrimp based on the highly abundant expression of *LvNHE* transcripts. Similarly, the gastrointestinal tract is one of the major sites of NHE expression in mammals, and the *NHE* cDNA was first isolated from the brush-border membrane vesicles in the small intestine and kidney of rat [18].

Previous studies have shown that incubation in acidic medium may increase *NHE* expression in mammalian cells, for example, the *NHE-3* mRNA expression in opossum kidney (clone P, OKP) cells [37]. In the euryhaline marine fish *Fundulus heteroclitus*, significant increases in both NHE1 and NHE3-like protein levels were induced by environmental hypercapnia [38]. In our current study, an inducible expression of intestinal *LvNHE* was detected upon low pH challenge. The upregulated *LvNHE* expression in the medium with a pH lower than 7.2 could last from 2 h to 6 h, suggesting that *LvNHE* was sensitive to a relatively low pH stress. Very interestingly, the up-regulation of *LvNHE* mRNA did not follow an acidity-dependent manner, and the maximal response of this gene was activated by pH 7.0. Based on the fact that the acidity/alkalinity of cells is mediated corporately by multiple ion transporters [39, 40], we speculate that the when the pH further declines, more pH-regulatory ion transporter genes may participate to prevent a further acidification of the cells, and the response and effect of NHE may be reduced.

It is generally believed that epithelial cells in the gut, antennal glands, integument and gill of crustaceans mediate the ion transportation into and across the tissues and thereby influence the concentrations of ions in the hemolymph [41]. In this study, we knocked down the *LvNHE* mRNA levels in shrimp by the RNAi approach and placed shrimp in an extreme environment of pH 5.8 to determine the effect of *LvNHE* on the regulation of circulating pH.

The hemolymph pH was measured in shrimp with/without *LvNHE* RNAi 6 and 12 h after a transfer from pH 8.0 to pH 5.8 seawater. At 6 h after the transfer to pH 5.8, the hemolymph pH values in shrimp without injection or shrimp injected with DEPC-H_2_O and NC-siRNA were highly comparable to that in shrimp kept at pH 8.0. However, the hemolymph pH in shrimp injected with *LvNHE* siRNA-2 was significantly lower than in the other groups, indicating that shrimp could not maintain the stability of hemolymph pH in acidified conditions after silencing of *LvNHE* expression. A similar phenomenon was observed in shrimp transferred to pH 5.8 for 12 h. Very interestingly, a slight increase appeared in shrimp transferred to pH 5.8 without RNAi of *LvNHE*. We speculate that there was an unknown physiological response initiated against the harmful acidified environment.

In the normal cells, where pH_i_ was reduced by acidification, the membrane ion transporters may pump out the protons for the cell realkalization [42]. To investigate the functional roles of *Lv*NHE in the cell realkalization after acidification in shrimp, the pH_i_ was detected in the intestinal fragment cultures from shrimp with/without knockdown of *LvNHE* transcript expression. BCECF-AM is a nonfluorescent membrane-permeating acetoxymethyl ester, which can be introduced into the cell where it is easily cleaved by nonspecific esterases into highly fluorescent membrane-impermeable BCECF. Loading with the BCECF-AM ester and subsequent concentration of de-esterified dye within the cell depend critically on the integrity of intracellular enzymes and of the cell membrane. In a damaged cell, after cleavage of the ester bonds, the hydrophilic BCECF would diffuse rapidly out of the cell, so it is used as an indicator of pH in living cells [31]. The basal pHi of shrimp intestinal cells was 7.26±0.054 (Fig 5B), significantly lower than the circulating pH. Similarly, the intracellular pH has been reported to be ~0.3 higher than the extracellular pH in humans, rainbow trout and the crab *Cancer pagurus* [43]. The NH_4_Cl perfusion is a classic method for rapid acidification of cells [31], and it was applied in this study. Cells were incubated in the absence of Na^+^ solution containing NH_4_Cl, leading to cellular accumulation of NH_4_^+^. Upon a subsequent shift to an NH_4_Cl-free solution, cellular NH_4_^+^ ions left the cells in the neutral NH_3_ form, leaving H^+^ behind, thus resulting in cytoplasmic acidification [44]. The recovery rate of pH_i_ in the *LvNHE* siRNA-2-injected group was much lower than in the control group, indicating that *LvNHE* participates in the process of cell realkalization after acidification. The acidification of pH_i_ is normally caused by increases in pCO_2_ [45], exposure to NH_4_^+^ and subsequent removal [28], and acid exposure [46]. The acidified intracellular conditions may give rise to a decrease in Na^+^ conductance and loss of excitability [47], loss of electrical coupling in early embryonic cells [48] and decreased light sensitivity in invertebrate photoreceptors [49]. Therefore, realkalization is necessary for cells to survive after they are acidified. Previous studies have reported the involvement of the Cl^-^/HCO_3_^-^ exchanger (AE) [46], V-type H^+^-ATPase [28] and NBC [50] in pH_i_ recovery in mammals. The mechanism of cell realkalization has not yet been developed in *L*. *vannamei*, and our present study is the first to provide the evidence for *LvNHE* participation in realkalization of pH_i_ in the *L*. *vannamei* intestine.

In summary, we first identified and characterized a full-length cDNA of the *NHE* gene from *L*. *vannamei* and analyzed the tissue expression profile and intestinal subcellular localization of *LvNHE* mRNA. The functional roles of *Lv*NHE in pH regulation of *L*. *vannamei* were demonstrated with the evidence that 1) the intestinal *LvNHE* mRNA levels increased under the low pH challenge conditions, 2) knockdown of *LvNHE* mRNA may reduce the hemolymph pH in shrimp under extreme low pH conditions, and 3) knockdown of *LvNHE* mRNA may reduce the realkalization capacity of intestinal cells when they are acidified. Altogether, this study sheds light on the regulatory effects of the NHE on extracellular (circulating) and intracellular pH regulation of *L*. *vannamei*. Our findings also provide the basis for protecting cultured *L*. *vannamei* from acidified aquatic environments.
